# Benchmarking of methods to analyse data derived from GBS-MeDIP

**DOI:** 10.1186/s12859-025-06330-x

**Published:** 2026-01-19

**Authors:** Violeta de Anca Prado, Fábio Pértille, Pedro Sá, Marta Gòdia, Joëlle Rüegg, Josep C. Jimenez-Chillaron, Carlos Guerrero-Bosagna

**Affiliations:** 1 Physiology and Environmental Toxicology Program, Department of Organismal Biology, Uppsala University, Uppsala, Sweden; 2 Animal Breeding and Genomics, Wageningen University & Research, Wageningen, The Netherlands; 3 Department of Physiological Sciences, School of Medicine, University of Barcelona, Barcelona, Spain

**Keywords:** DNA methylation, Genotyping by sequencing, Methylated DNA immunoprecipitation, Benchmarking, Pipeline

## Abstract

**Background:**

Genotyping-by-Sequencing with Methylated DNA Immunoprecipitation (GBS-MeDIP) is an emerging method for cost-effective DNA methylation analysis. However, due to its unique sequencing output, conventional bioinformatics pipelines used for RNA-seq and MeDIP-seq are not fully adequate for analyzing GBS-MeDIP data. Selecting the appropriate statistical methods for differential methylation analysis remains a challenge, as existing approaches may introduce bias or false positives.

**Results:**

We benchmarked multiple statistical methods for analyzing GBS-MeDIP data using previously generated datasets from chickens, dogs, and pigs. FeatureCounts was identified as the most reliable tool for count matrix generation, outperforming MEDIPS, which introduced biases in count estimation. For differential methylation analysis, we evaluated EdgeR, limma, DESeq2, and the Mann-Whitney test. Our results demonstrated that Mann-Whitney provided the lowest false positive rate and highest true positive rate, outperforming both EdgeR, DESeq2, and limma. EdgeR’s quasi-likelihood method exhibited a high false positive rate, making it unsuitable for GBS-MeDIP analysis.

**Conclusions:**

Our findings highlight that GBS-MeDIP data should not be analyzed using standard RNA-seq or MeDIP-seq pipelines, as these approaches lead to statistical artifacts. Instead, we recommend featureCounts for count matrix creation and Mann-Whitney for differential methylation analysis, ensuring accurate detection of differentially methylated windows. This study provides a bioinformatics framework for analyzing GBS-MeDIP data, minimizing biases and improving reliability in epigenomic research.

**Supplementary Information:**

The online version contains supplementary material available at 10.1186/s12859-025-06330-x.

## Background

High-throughput sequencing technologies have become more cost-efficient over time, significantly reducing the price per sample. This reduction allows for higher number of sample sizes, increasing statistical power in further analyses.

 Genotyping-By-Sequencing (GBS) was developed to enable researchers to genotype many individuals and improve the reliability of population genomic studies [1]. This method has been used in plant and animal breeding for over 20 years [2] as a reliable tool to study diversity, perform genome-wide association studies (GWAS), conduct general population genomics research in a variety of taxa [3, 4], and elucidate genetic relationships in farm animals [5]. The method involves enzymatic digestion of the DNA followed by size selection to obtain a reduced representation of the genome, thus enabling the genotyping of populations in a cost-effective manner [3].

Beyond genomics, studying DNA methylation, one of the most study epigenetic modifications for its impact on gene expression regulation, is of great interest to the research community. Whole Genome Bisulfite-sequencing (WGBS) is the gold-standard method for methylomic analysis. It involves the conversion of all non-methylated cytosines to uracil using bisulfite treatment, which are then recognized as thymines upon amplification. Although WGBS offers single base resolution, the significant nucleotide imbalance that is produced poses a major challenge [6]. Another disadvantage of this method is its high cost, as a large portion of the data sequenced can be uninformative [7]. As the need for cost-effective methods is of great interest, various options to study genome methylation have been developed. This includes Reduced Representation Bisulfite Sequencing (RRBS) [8], which examines methylation patterns in targeted regions in the genome. This protocol uses bisulfite conversion on DNA digested by a methylation-sensitive restriction enzyme, and is by design biased against higher cytosine-guanine dinucleotide (CpG)-dense regions of the genome (≥ 3 CpG/100 bp) [9, 10]. While RRBS is used on a wide range of species, an alternative method popular for its simplicity, both in analysis and interpretation, is the Illumina BeadChip array (either Infinium or the HumanMethylation450 BeadChip) [11]. This method consists of a set of predefined location across the genome where bisulphite converted DNA attaches to probes. Highly used in clinical research, this method is consistent but lacks species diversity and is subjected to fixed number of locations in a similar manner as genotyping, where new variations are not taken into account.

An alternative non-bisulphite conversion method is Methylated DNA immunoprecipitation followed by sequencing (MeDIP-seq), where the DNA is fragmented via sonication and then immunoprecipitated with antibodies against 5-mehtyl cytosine (5mC) [12]. However, whole genome MeDIP-seq is also costly, thereby limiting its use for a large number of samples. A recently published method, GBS-MeDIP [13], provides a cost-effective solution by combining features of both GBS and MeDIP. Briefly, in this method the DNA is fragmented with the enzyme *PstI*, barcoded, pooled and then the pooled barcoded DNA is immunoprecipitated with 5mC-antibodies. Therefore, immunoprecipitation is performed on a pool of samples and not on individual samples. Advantages of the GBS-MeDIP include its cost effectiveness due to the use of reduced genomes and inclusion of many individuals in sequencing libraries. Another advantage is the ability to investigate genetic and methylomic variants in the same genomic regions, as the methylomic library is the immunoprecipitated pool that is originated from the genetic library. This advantage is crucial in multigenerational and transgenerational projects, where there is an interest on obtaining the same genomic regions across individuals and generations. Another advantage of this method is that there is no bias against CpG islands, but it has been noticed an enrichment in repeated elements (RE), as usually RE are systematically silenced by methylation [14]. One potentially drawback in GBS-MeDIP is that it can only be used in comparison studies, and as GBS, can suffer from polymorphisms around the restriction enzyme when comparing different populations. Nevertheless, GBS-MeDIP produces a library that is count-based, meaning that the level of methylation is assumed to be proportional to the number of reads assigned to that specific region. Because of the uniqueness of the sequencing output, which includes stacked reads of fix regions due to the enzymatic cut from *PstI*, the bioinformatic pipelines developed to analyse sequencing data generated by other techniques, such as gene expression or MeDIP, are not fully adequate for data generated via GBS-MeDIP. In the present paper, we investigate different options to optimize the pipeline to analyse GBS-MeDIP data using both simulations and publicly available data.

In count data analysis, the two critical steps are (i) creating the count matrix and (ii) performing differential statistical analysis. Counts can be assigned in two different ways: by using a pre-existing list of genomic features, as it is done in RNA-seq, or by defining the window of interest within the library based on the location of peaks of reads as performed in the Assay for Transposase-Accessible Chromatin (ATAC-seq), Chromatin Inmunoprecipitation-sequencing (ChIP-seq) and MeDIP-seq. Once the count matrix is created, differential analyses can be conducted. Table 1 summarizes the statistical methods used for differential analysis of RNA-seq, MeDIP-seq, and other related statistical tests. The listed methods were chosen due to their established use in biological count data analysis or because they require minimal statistical assumptions.

**Table 1:** Summary of all the methods considered to perform statistical differences on GBS-MeDIP data

Method	*R* package	Author	Number of citations	Distribution	Test statistic	Input data
Maximum likelihood	EdgeR	[15]	31,395	Negative binomial	Likelihood ratio test	RNA-seq/MeDIP-seq
Quasi likelihood	EdgeR	[16]	706	Negative binomial	Likelihood ratio test	RNA-seq
Moderated t-test	limma	[17]	769	Not applicable	Moderated t-test	RNA-seq
Mann-Whitney	Stats	[18]	15,127	Not applicable	Mann-whitney	Applied to many biology fields
DESeq2	DESeq2	[19]	84,039	Negative binomial	Likelihood ratio test	RNA-seq

EdgeR, DESeq2 and limma are software widely used for analyzing count data derived from short-read sequencing. EdgeR fits data into a negative binomial distribution using different approaches, depending on the data dispersion. It uses General Linear Models (GLM), where the null hypothesis represents a simpler model than the alternative hypothesis [15]. In a case-control study design, the null hypothesis posits that there is no difference between the two groups. The DESeq2 package also utilizes GLMs and the negative binomial distribution. The limma package approach for read count normalization is based on logarithmic conversion to counts per million (log2CPM) followed by differential analysis through moderated t-test, where the standard error is taken from all input genomic locations. The null hypothesis for the moderated t-test is that there are no differences between the means [17]. The Mann-Whitney U-test is also a suitable approach since it is a non-parametric test, thus with less assumptions on the data distribution. Mann-Whitney compares the ranks distributions between groups, with the null hypothesis stating that there are no differences between them, thus it is the non-parametric equivalent of the t-test for comparing the mean [18].

In this article, we present a benchmark of count and statistical methods for analysing GBS-MeDIP data using simulated data and previously generated datasets from three different animal models: chickens [20], dogs [21], and pigs [22]. Despite the fact that GBS-MeDIP data have features that resemble data derived from RNA-seq, we showed that GBS-MeDIP data cannot be analysed with methods designed for RNA-seq nor MeDIP-seq. Based on our analyses, we have created an optimized pipeline to analyse GBS-MeDIP generated data.

## Methods

### GBS-MeDIP data

Publicly available datasets from GBS-MeDIP were retrieved from the European Nucleotide Archive (ENA). The datasets included four different species: chicken, with comparisons of the same breed (White Leghorn) in two different environments (total *N* = 12) (ENA ID: PRJEB34868) [20]; dog and wolf, with comparisons of their epigenome (total *N* = 6) (ENA ID: PRJEB32791) [21]; and pig, with comparison of Landrace and Large White breeds (total *N* = 26) (ENA ID: PRJEB43108) [22]. The data were downloaded as bam files, thus the sequencing reads were already mapped to the chicken (Gallus_gallus 5.0) [23], dog (CanFam3) [24] and pig (Sus scrofa 11.1) [25] genome, respectively, using Bowtie2 v.2 [26] with default parameters. All bam files were filtered for mapping quality MQ >10 with samtools v.1.14 [27]. The bam files were merged, and the genomic coordinates of all concordant pair of reads were extracted. The mean length of the extracted genomic coordinates was calculated, and windows exceeding 300 bp were subset and fragmented to the mean length. This step ensures that no window exceeds the mean length of the library, as resolution would be compromised. All windows were then merged and ordered in a SAF format (tabulated table including chromosome, start, end position and an identifier) using a custom script.

### Count matrix assessment

To test the performance of the different count methods the generated saf files were queried against the filtered bam files using three different software. First, the MEDIPS.meth from the R package MEDIPS v1.52.0 [28] was tested. This package was used only in the windows defined by the SAF file previously created, by using paired-end mode and the parameter uniq set to 0. The reason for this is that with the GBS-MeDIP method the start and end site are consistently positioned, due to its unique enzymatic cut site (5′-CTGCA/G-3′). The second approach tested was performed using featureCounts v2.0.3 [29]. The SAF file was used as input, and flags were set for paired-end reads, proper alignment, and fragment length ≤ 1500 bp. In both cases, a count matrix was created, with individuals as columns and windows of interest as rows. These two software were then compared to bedtools multicov, a function from the BEDTools software [30] that calculates coverage in a certain region provided a bed file. Raw counts from each method were plotted against the bedtools output using ggplot2 v.3.5.1 [31] in R v.4.3.1.

### GBS-MeDIP window distribution

In order to assess the most appropriate statistical distribution of the windows from all datasets, as both MEDIPS and EdgeR use a negative binomial distribution, the Akaike Information Criterion (AIC) was computed [32] for several distributions: normal, poisson and negative-binomial on all windows from the three datasets on the non-normalized counts. To evaluate whether the distribution was preserved after normalization, read counts were adjusted by effective library size. This method corrects for read depth per individual, followed by the application of the trimmed mean of M-values (TMM). The TMM factors are multiplied by the library size, and the raw counts are divided by the resulting value to obtain normalized counts. Afterwards the AIC was computed for several discrete and continuous distributions: normal, poisson, negative-binomial, uniform, and logistic on all windows from the three datasets. This two calculations were done using the R package fitdistrplus v.1.1–11 [33]. The AIC values for both tests were visualized in Fig. 2 using ggplot2 in R.

### Statistical method performances

To assess if the statistical methods fit the data correctly, null distributions of p-values were created for all the statistical methods considered, as p-values derived from comparisons of a random distribution are expected to have equiprobability, thereby generating a uniform theoretical distribution. Using the count matrix of all datasets, we ran 1,000 iterations subsetting 10,000 windows and randomizing the raw counts, to create a random dataset. Each subset ran all the methods. EdgeR, limma and DESeq2 have a normalization step before performing differential analysis whereas Mann-Whitney was supplied with a normalized count matrix which was obtained following the same method EdgeR uses (*calcNormFactors*), and 10,000,000 p-values were extracted per running method. The null distribution of p-values was then visualized against the theoretical quantiles of a uniform distribution using ggplot2 in R and their density was represented on bins representing 1% of the data from 0 to 1. After the False Positive Rate (FPR) [34] was calculated for each method in each dataset.

To calculate the True Positive Rate (TPR) [35], and Receiver Operating Characteristic (ROC) curves [35], a simulated dataset was created to reflect the unique characteristics the wet lab method has, such as, windows with high, medium and low coverage, and windows that are highly-, low-, and non-differentially expressed. Under the negative binomial distribution, 17,100 windows were created for 10 individuals mimicking high-, low-, and non- Differentially Methylated Regions (DMRs) based on the different percentages of methylation, expressed in the mean (mu) parameter in the negative binomial distribution. High DMRs comprised of 8,100 windows, divided into sections with fold change (i.e., ratio between probabilities between each of the groups) 7, 2.5 and 1.23, low DMRs comprised of 7,200 windows, divided into sections with fold change 0.96, 0.8 and 0.62, and non DMRs comprised of 1,800 windows, divided into sections with fold change 0.43, 0.2 and 0.13. The different fold changes were tested across several coverages: 0.1, 0.5, 0.7, 0.9, 1,5, 10, 20 and 50. Coverage was modeled using a normal distribution to provide the dispersion parameter in the negative binomial model. After the count matrix had been created, each model was run and the p-values were collected. Benjamini-Hochberg and Bonferroni multiple test correction methods were applied in parallel. A confusion matrix was generated for each model with each multiple test correction method. In the confusion matrix, adjusted p-values less than 0.05 from the high and low DMR groups were considered as correct, as well as adjusted p-values above 0.05 if they belonged to the non DMR windows. Visualizations were created with ggplot2 in R.

## Results

To correctly assess differential methylation in any study, it is crucial to ensure that the count matrix is constructed properly and that counts reflect the reality of the sequenced library. For the datasets used in this study, the remaining number of mapping reads with MQ > 10 were as follows: for the chicken dataset, a total of 22.83 M reads were obtained, with average of 3.97 M; for the pig dataset a total of 7.51 M reads were obtained, with average of 0.57 M; for the dog and wolf dataset, a total of 15.54 M reads were obtained, with average of 5.18 M. Supplementary Table 1 provides detailed information on the average reads per individuals.

### Count matrix performance

Based on the reads of these datasets, we performed the benchmarking of tools to create the count matrix, with the objective of performing differential methylation analysis to accurately represent each covered window. MEDIPS and featureCounts were compared against BEDTools, which generates coverage of regions outputs. While BEDTools does not have any filter, the comparison with featureCounts mostly aligns in a diagonal, thus showing that featureCounts reports accurate counts. Contrastingly, the comparison with MEDIPS showed counts either higher or lower than the actual coverage of the library (Supplementary Fig. 1). While MEDIPS involves a fitting of the counts with a poisson distribution, featureCounts does not involve any fitting of the counts. Instead, featureCounts filters for all reads properly aligned in pair end mode, which can be observed on the few discrepant windows. Since featureCounts effectively handled the type of data produced by GBS-MeDIP, it was chosen as the most fitting count tool. A total of 209,452, 56,694 and 99,328 windows were generated for the chicken, dog, and wolf, and pig datasets, with assigned 1.43 M, 1.49 M, and 0.27 M reads on average, respectively. Supplementary Tables 3, 4, and 5 shows specific information on the number of assigned reads per individual for each of the datasets.

### Nature of GBS-MeDIP windows

**Fig. 1:**
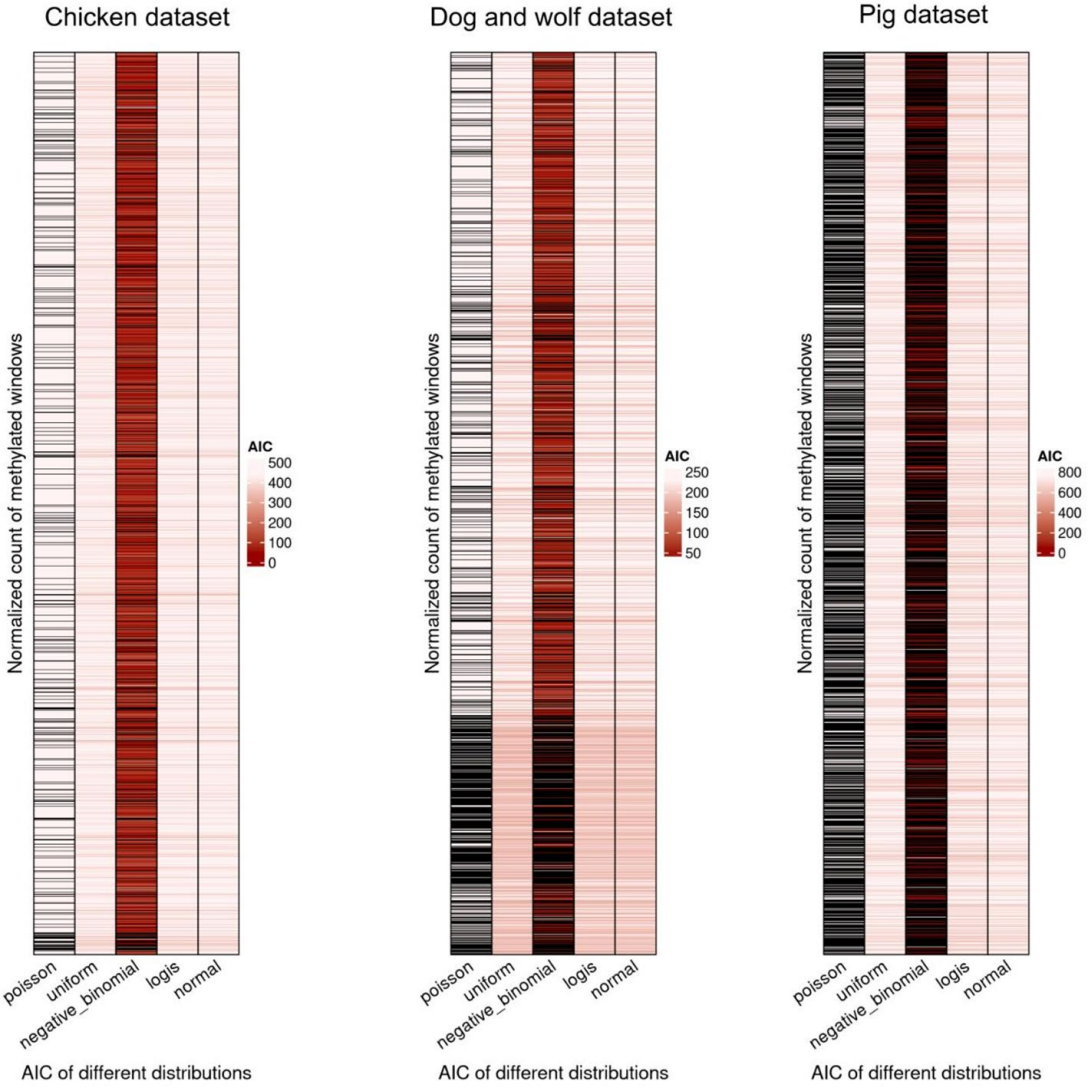
AIC representation in all read count normalized windows from the three datasets collected for this study. In red is represented the most significant AIC for the distributions compared: normal, poisson, negative-binomial, uniform and logistic. With the colour black is represented those windows which broke assumptions for the pertained distribution. Across all datasets the negative binomial distribution is the one with the most significant AIC (the lowest). Note that there are a significant number of windows where the assumptions for the negative binomial distribution are not met

With the count matrix established using featureCounts, the next step involved evaluating how statistical models fit the data to assess differential methylation. EdgeR, DESeq2 and MEDIPs attempt to fit the data into a negative binomial distribution to later on assess significance. Data that do not follow a negative binomial distribution cannot be assessed for DMR with those methods, as it would lead to bias. In order to check the distribution of the GBS-MeDIP data, the non-normalized windows were tested for the fitting to poisson, negative binomial and normal distributions using the AIC, which is a relative measure of the fitness between the compared distributions. The negative binomial distribution turned out to be the best fitting distribution for the majority of the windows (91.90% for the chicken dataset, 91.10% in the dog and wolf dataset and 88.72% in the pig dataset) (Supplementary Fig. 2). This distribution models a generalization of the times in our libraries where a methylated CpG is encountered. It is not expected to, nonetheless, the distribution can change when read count normalizing data. For this, all read count normalized windows from all datasets were tested again using the AIC. The AICs for most of the known distributions (normal, poisson, negative-binomial, uniform and logistic) are shown in Fig. 1. The majority of the read count normalized windows can be fitted into a negative binomial distribution, as the most significant AIC for the majority of the datasets is the negative binomial distribution (85.64%, 64.04% for the chicken and dog and wolf dataset, respectively). However, a number of windows break the assumptions of this distribution, one example being the pig dataset in which the majority of the windows have a lower AIC following the logistic distribution. This creates a difficulty in choosing MEDIPS or EdgeR as their read count normalization would lead to bias in the analysis.

### Statistical method performances

**Fig. 2:**
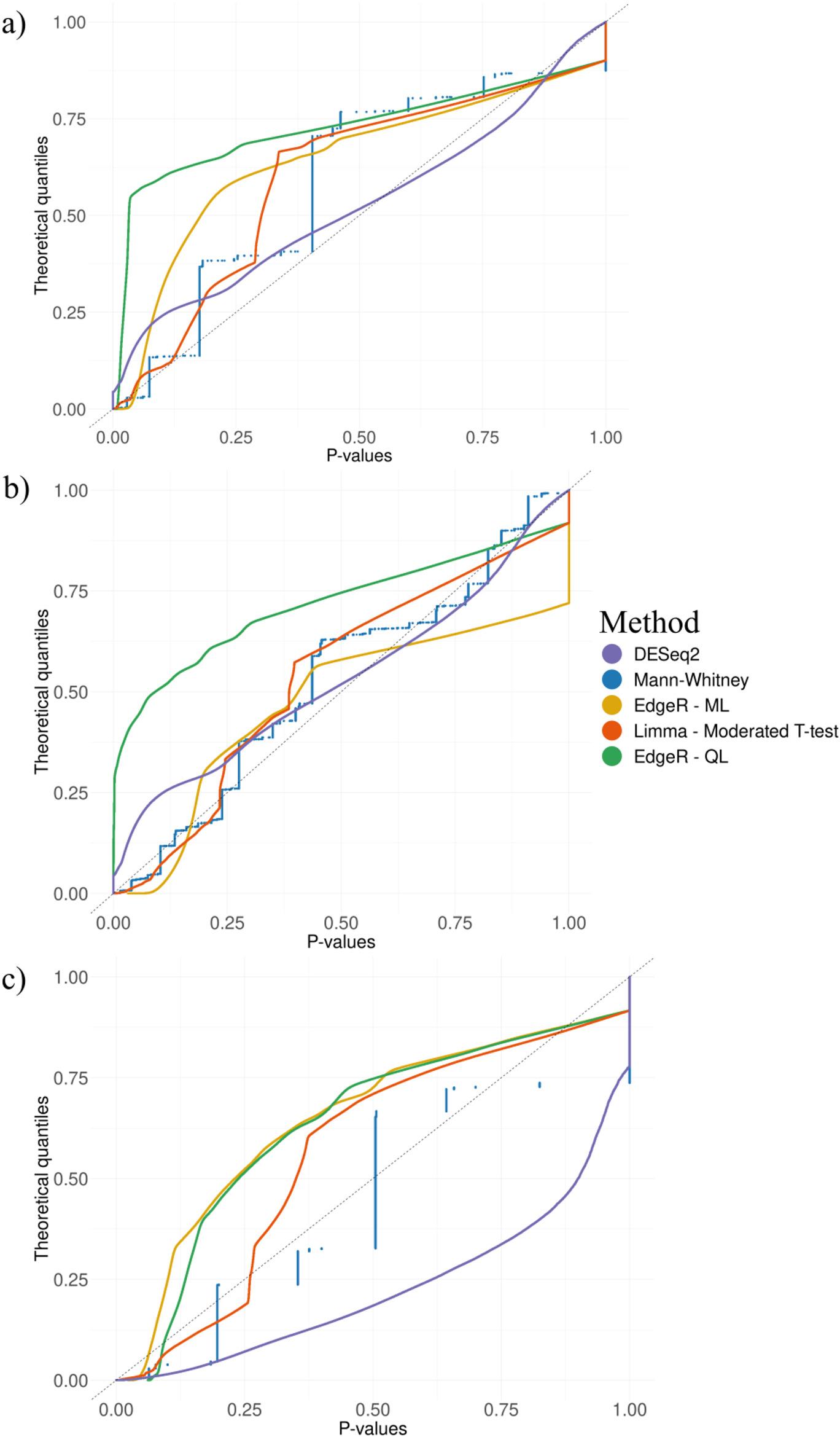
P-value uniformity test of Mann-Whitney, EdgeR using Maximum-likelihood (ML), EdgeR using Quasi-likelihood (QL), DESeq2, and limma. 1,000 iterations of 10,000 randomized windows from each of the datasets used in this study were tested. 10,000,000 reported p-values were plotted against the theoretical quantile. **A** P-value uniformity test of a random distribution of *N* = 12 using the chicken dataset, **B** P-value uniformity test of a random distribution of *N* = 26 using the pig dataset, **C** P-value uniformity test of a random distribution of *N* = 6 using the dog and wolf dataset

Building on this initial evaluation of data fitting into statistical distributions, the next step involves assessing the performance of the methods by examining the uniformity of p-value distributions derived from randomization of counts to ensure their reliability in identifying DMRs. For this, p-values were generated for 10,000 windows over 1,000 iterations for the datasets and plotted against the quantiles of a uniform distribution (0,1). The expectation for an unbiased method is that the p-values would lie on the diagonal. Figure 2 shows the Mann-Whitney test displayed a uniform distribution, although an increased staggering of observed p-values was noted. However, the distribution of null p-values generated by EdgeR using maximum likelihood and quasi-likelihood, and by limma, did not adhere to the diagonal, therefore representing a biased distribution. DESeq2 displays very little bias where the number of individuals are bigger than 10, this can also be noted in Supplementary Fig. 2, were the density of p-values derived from each method can be observed in 1% bins.

For random datasets of methylated counts, approximately 5% of false positives (p-values lower than 0.05) are expected. A method that handles correctly the data should adhere to this rate. Using the same random datasets, we calculated the FPR. The average FPR values across datasets were: 2.13% for Mann-Whitney; 2.53% for EdgeR using maximum-likelihood, 33.26% for EdgeR using quasi-likelihood, for DESeq2 11.64%, and 3.28% for limma using the moderated t-test. The individual FPR values of each dataset employing different methods are shown in Supplementary Table 2. The different sample sizes (N) in each dataset affected the statistical power in all the models. For example, in the dog and wolf dataset with *N* = 3 per group, the FPR was near 0 or very low for each method. EdgeR with maximum likelihood also produced FPR below 5%, yet, as seen in Fig. 2, displayed a bias. The quasi-likelihood method in EdgeR produced a high number of false positives. DESeq2 displayed very high FPR but adhered to the diagonal the best. Lastly, limma was the second-best performing method with a FPR (3.28%) very close to that of Mann-Whitney, the top performer.

In addition to observing the FPR for each method, it is also important to determine which method has sufficient power for a high TPR, which entails assurance of obtaining true DMRs when analysing GBS-MeDIP data. As the wet lab method does not have a validated dataset, a simulation was devised with window counts for high DMRs, low DMRs and no DMRs. Each of the methods were also compared using the two most commonly used multiple test correction methods, namely Benjamini-Hochberg (FDR) and Bonferroni. With either correction, Mann-Whitney, had a TPR over 76%, limma and DESeq2 performed intermediately with a TPR of 66% and 70% respectively, whereas EdgeR performed below 66%. Table 2 provides a detailed description of each individual method with FDR and Bonferroni correction.

**Table 2:** True positive rate (TPR), on simulation of 17,100 windows by Mann-Whitney, edger (Maximum likelihood and Quasi-likelihood), DESeq2, and Limma (Moderated t-test), calculated for two multiple test Correctoin test, Benjamini-Hochberg (FDR) and Bonferroni

	Maximum Likelihood with FDR test correction	Quasi-likelihood with FDR correction	Moderated t-test with FDR correction	Mann whitney with FDR correction	DESeq2 with FDR correction	Maximum Likelihood with Bonferroni correction	Quasi-likelihood with Bonferroni correction	Moderated t-test with Bonferroni correction	Mann whitney with Bonferroni correction	DESeq2 with Bonferroni correction
TPR	0.66	0.65	0.65	**0.92**	0.70	0.53	0.53	0.50	0.76	0.54

The TPR ranges from 0 to 1, 1 meaning the method does not have any false positive nor negative. Highlighted in bold is the hightest TPR which corresponds to Manny-Whitney using FDR as multiple correction test

**Fig. 3:**
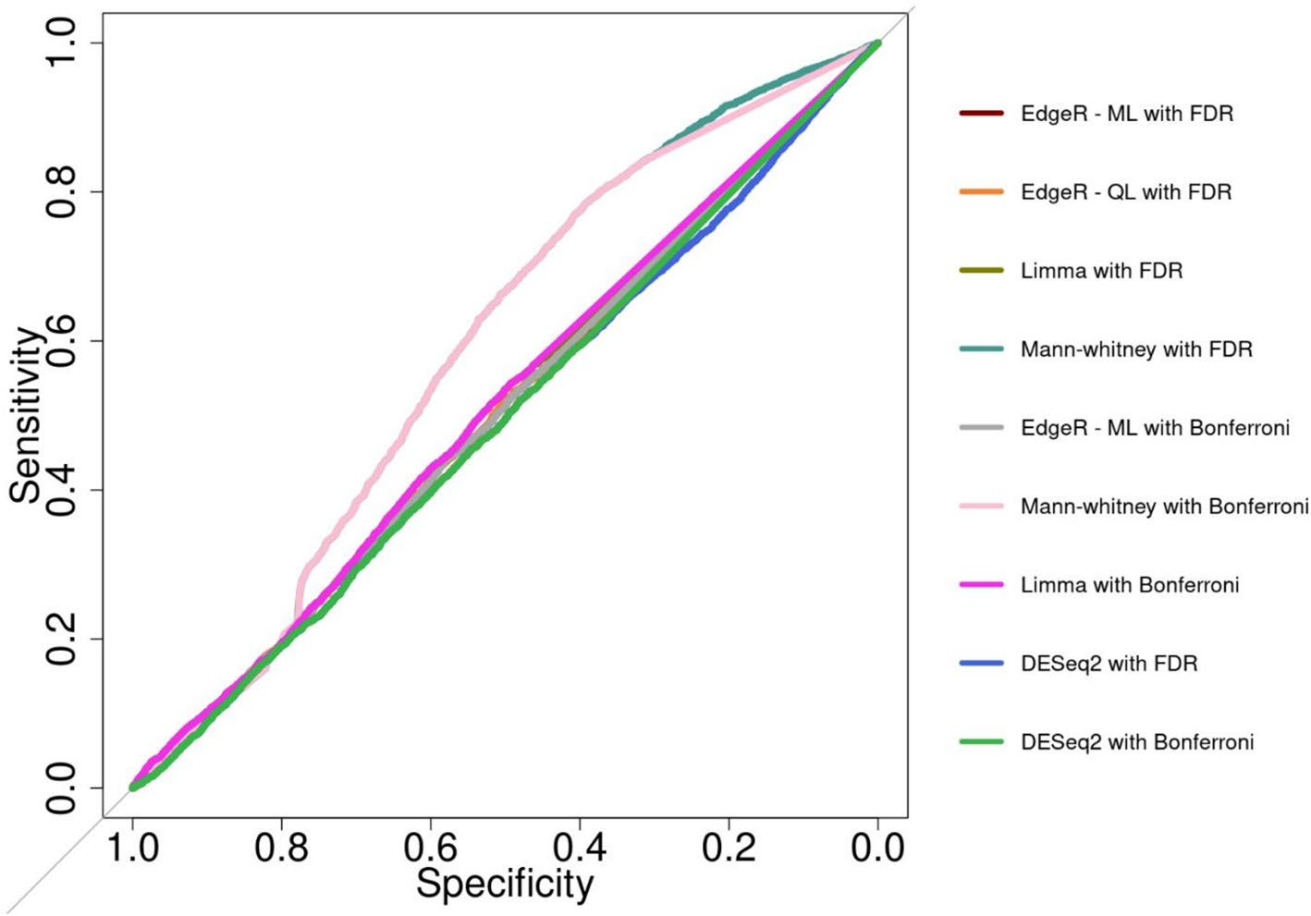
Receiver operating characteristic (ROC) curve. Each statistical method was tested with a simulation of 17,100 windows mimicking high DMRs, low DMRs, and not differentially methylated

Once the TPR values were calculated, the methods could be compared by their ROC curves (Fig. 3). The ROC curve is a visualization of the best method to detect DMR: the higher the area under the curve a method displays, the more accurate the method is. Figure 3 shows that Mann-Whitney displays a higher area under the curve than the rest of the methods tested. It is also interesting to note that Bonferroni and FDR display very similar pattern with all the methods. With a higher area under the curve, Mann-Whitney is shown to be the best discriminant method for detecting DMRs.

## Discussion

In this article, several methods to analyse data derived from GBS-MeDIP were benchmarked. This study also demonstrates that common statistical methods employed to perform differential methylation analysis based on RNA-seq analysis or MeDIP-seq are not suitable for GBS-MeDIP data, as they exhibit a high false positive rate.

The selected tool for creating the count matrix from data derived from GBS-MeDIP libraries must accurately represent each covered window. Therefore, two widely used count features employed in gene expression analyses and MeDIP-seq were compared in this article to determine which one accurately reports counts from GBS-MeDIP data. featureCounts is one of the most popular count feature tools available for differential gene expression analysis, while MEDIPS is the gold standard for analyzing MeDIP-seq data. MEDIPS failed to accurately report counts compared to BEDTools. To avoid exacerbating numbers from potential duplication obtained after the PCR procedure in the bench work, MEDIPS models the count with a poisson distribution. Due to this, MEDIPS appears to shrink the counts of some windows while exacerbating others, with no apparent pattern observed, making it unsuitable for GBS-MeDIP data analysis (Supplementary Fig. 1). The other count method evaluated, featureCounts, which does not involve any calculation of PCR duplicates, accurately reported counts across the different datasets. Only a small fraction of the counts diverged from the reporting of coverage of BEDTools, likely due to the advanced filtering featureCounts discarding bad quality or misaligned reads.

Complex statistical tests often involve fitting the data into a particular distribution after read count normalization. In this article, we evaluated the EdgeR and DESeq2 software, which follow this pattern when attempting to fit the data given into a negative binomial distribution for later assessment of significance. It might seem as the read count normalization step followed by EdgeR is altering the distribution of the data (Fig. 1), making it unsuitable for differential analysis using a negative binomial (Fig. 2 and Supplementary Fig. 3). On the other hand, it seems the read count normalization of DESeq2 suits better GBS-MeDIP data as the null p-value distribution is quite uniform, even if it encounters a high FPR. We have shown that GBS-MeDIP data do not follow a negative binomial distribution in the tested datasets after read count normalization from EdgeR. Thus, GBS-MeDIP data cannot be assessed for DMR with EdgeR methods, as it would lead to bias; currently there is no alternative of model fitting. Noteworthy is the observation that the non-normalized counts indeed follow a negative binomial distribution, which is relevant, as it is widely used in methylation data [36].

This study analysed the reliability of various statistical methods for detecting differential methylation in three different datasets spanning four species. To ensure a correct assessment of DMRs, the p-values obtained from a random set of methylated windows should be uniformly distributed uniformly. Any deviation suggests that the method is not appropriate to model the data correctly, or that the assumptions are not met. In this article, we show that presented how Mann-Whitney generates staggering p-values in the uniformity test (due to the discrete nature of its distribution) while still adhering to the diagonal in contrast to the bias that displayed the rest of the methods. DESeq2 also displayed a very uniform distribution, but the FPR produced by this method surpasses 15%. The Mann-whitey test outperformed in the TPR and was slightly conservative in the FPR, with a value of 2.13%, well below the 5% threshold. Even with a small sample size, the Mann-Whitney test did not show bias in the null distribution of p-values, but produced conservative p-values, concordant with being a non-parametric test. In contrast, EdgeR and limma had low TPRs (66% and 65% respectively), making them unreliable for analysing GBS-MeDIP derived data. The EdgeR quasi-likelihood method produced the highest FPR, likely due to the overdispersion parameter introduced by the quasi-likelihood method, which can lead to a high level of false positives, as overparameterization can occur when applied to certain data [36, 37]. Alternative methods to the ones investigated in this article have also been used in genomic data, for example permutation test [38]. Permutation test compares the observed data to a distribution generated by permuting the same data randomly. The main drawback encountered when applying this method to GBS-MeDIP data is that it requires the permutations to have the same distribution, which was not the case in the tested datasets across all the windows.

Similar type of benchmarking studies can be found for Illumina methylation arrays and RNA-seq [39, 40] and also focusing on the importance of identify and minimize statistical artifacts when possible [41, 42]. Surprisingly, those studies are not found for MeDIP-seq, one of the most widely employed method to investigate DNA methylation. Even though an immunoprecipitation is performed in the process of GBS-MeDIP, the results presented in this article cannot be applied to MeDIP-seq, as it involves DNA sonication, forming a completely different library distribution from what GBS-MeDIP creates.

## Conclusions

In conclusion, this study shows that bioinformatic analyses tailored to MeDIP-seq or RNA-seq data should not be employed to analyse GBS-MeDIP generated data, as this will most likely result in false positives or casual relations. This study also shows that the best performing tools to analyse GBS-MeDIP derived data is featureCounts for count matrix creation and Mann-Whitney for differential analysis.

## Supplementary Information

Supplementary Material 1

Supplementary Material 2

## Data Availability

The scripts generated during the current study are available in the Violeta-de-Anca/Benchmarking-of-methods-to-analyse-data-derived-from-GBS-MeDIP.git repository, https://github.com/Violeta-de-Anca/Benchmarking-of-methods-to-analyse-data-derived-from-GBS-MeDIP. All the dataset used in this article are from previous published peer-reviewed articles and the ENA repository IDs are: PRJEB34868, PRJEB32791 and PRJEB43108.

## Abbreviations

GBS: Genotyping-by-sequencing
CpG: 5′–Cytosine–phosphate–Guanine–3′
PCA: Principal component analysis
GWAS: Genome-wide association studies
RRBS: Reduced representation bisulphite sequencing
MeDIP-seq: Methyl immunoprecipitation-sequencing
GBS-MeDIP: Genotyping-by-sequencing and methyl-immunoprecipitation
FPR: False positive rate
DMRs: Differentially methylated regions
ROC: Receiving operation curve
WGBS: Whole genome bisulphite sequencing
5mC: 5-Methyl cytosine
RE: Repeated elements
ATAC-seq: Assay for transposable-accessible chromatin
ChIP-seq: Chromatin inmunoprecipitation-sequencing
GLM: General linear model
AIC: Akaike information criterion
TPR: True positive rate
ML: Maximum-likelihood
QL: Quasi-likelihood
log2CPM: Logarithmic counts per million
