# Supplementary material for: Benchmarking of methods to analyse data derived from GBS-MeDIP
Source: BMC Bioinformatics. 2026 Jan 19;27:17. doi: 10.1186/s12859-025-06330-x (PMC12829230; doi:10.1186/s12859-025-06330-x)
Supplement: Supplementary file 2 — Supplementary Material 2 [file 12859_2025_6330_MOESM2_ESM.docx]

# Supplementary Figures


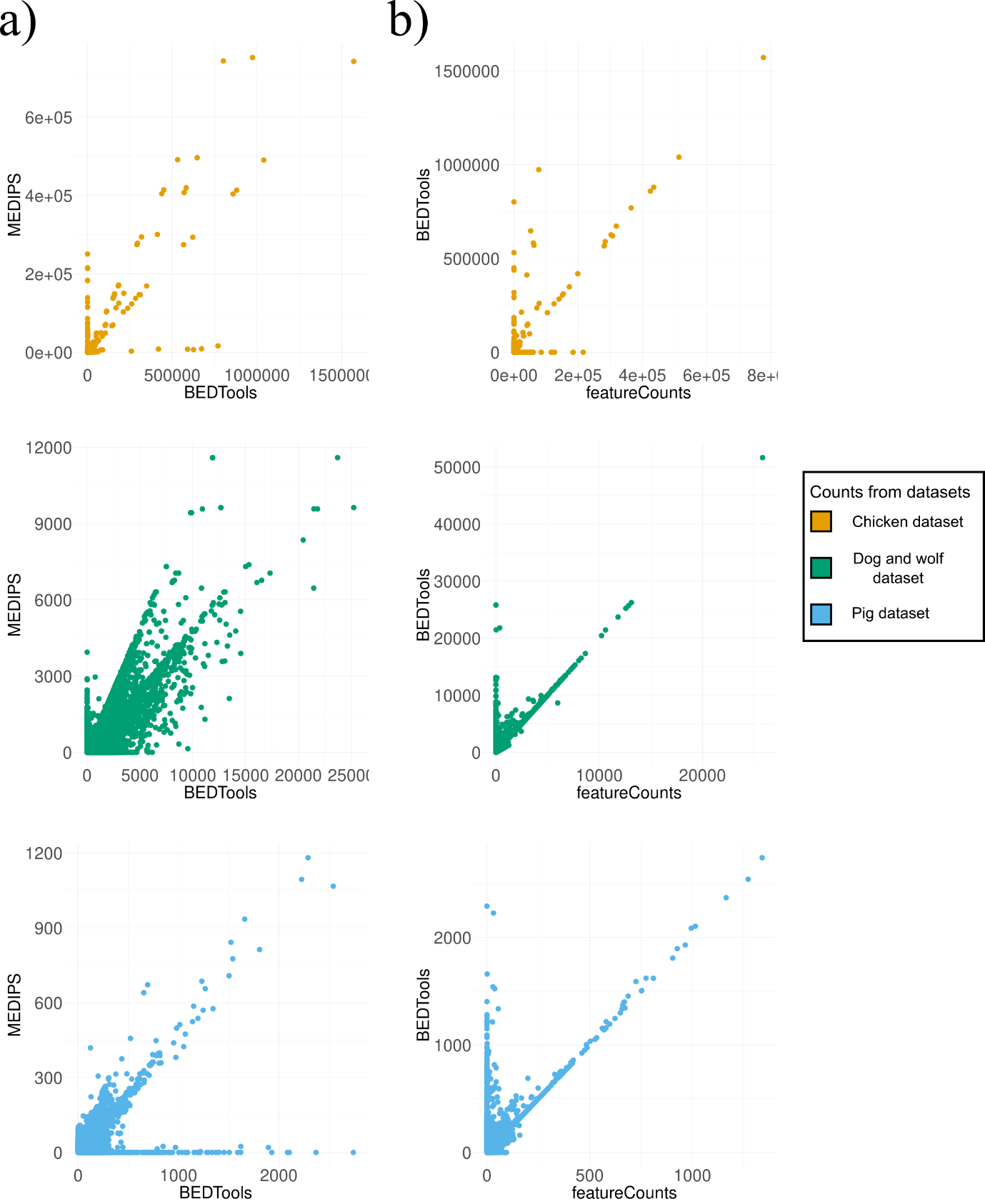


Supplementary Figure 1. Representation of the correlation between the count number reported by BEDTools and MEDIPS, and by featureCounts and BEDTools. If there was a complete report of counts between the two methods, we would see a perfect diagonal. a) It can be observed that for some windows MEDIPS exacerbate the counts (meaning for that window there is a higher number than what it is sequenced) or lowers the count number (MEDIPS reports a lower number than what is sequenced). b) featureCounts is set to filter out and not report fragments that are not properly aligned on both reads, creating a discrepancy as it sets to 0 or lower counts windows were there is coverage as seen by BEDTools reports.


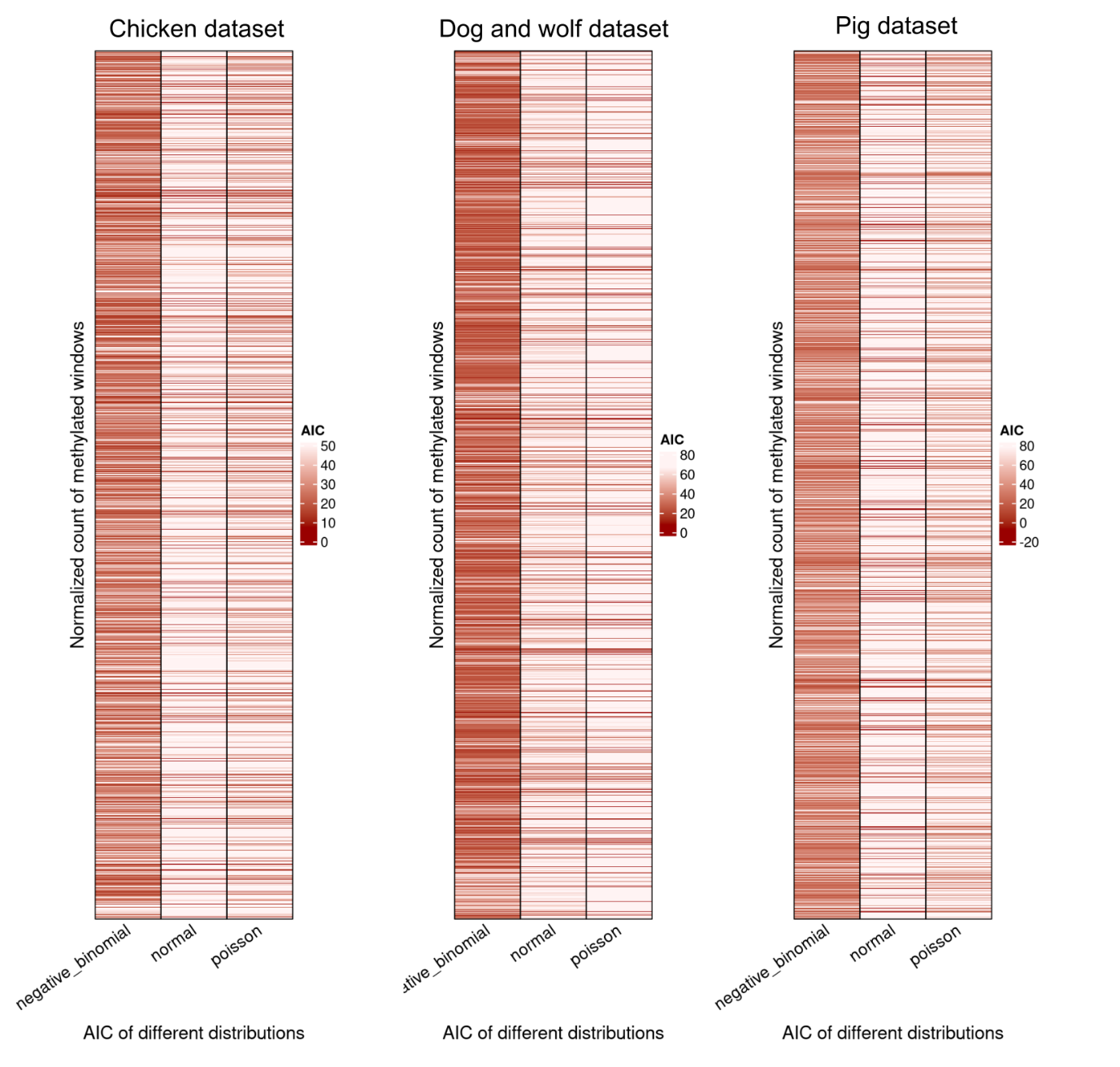


*Supplementary Figure 2. AIC representation in all non-normalized windows from the three datasets collected for this study. In red is represented the most significant AIC for the distributions compared: normal, poisson, and negative binomial. Across all datasets the negative binomial distribution is the one with the most significant AIC (the lowest).*


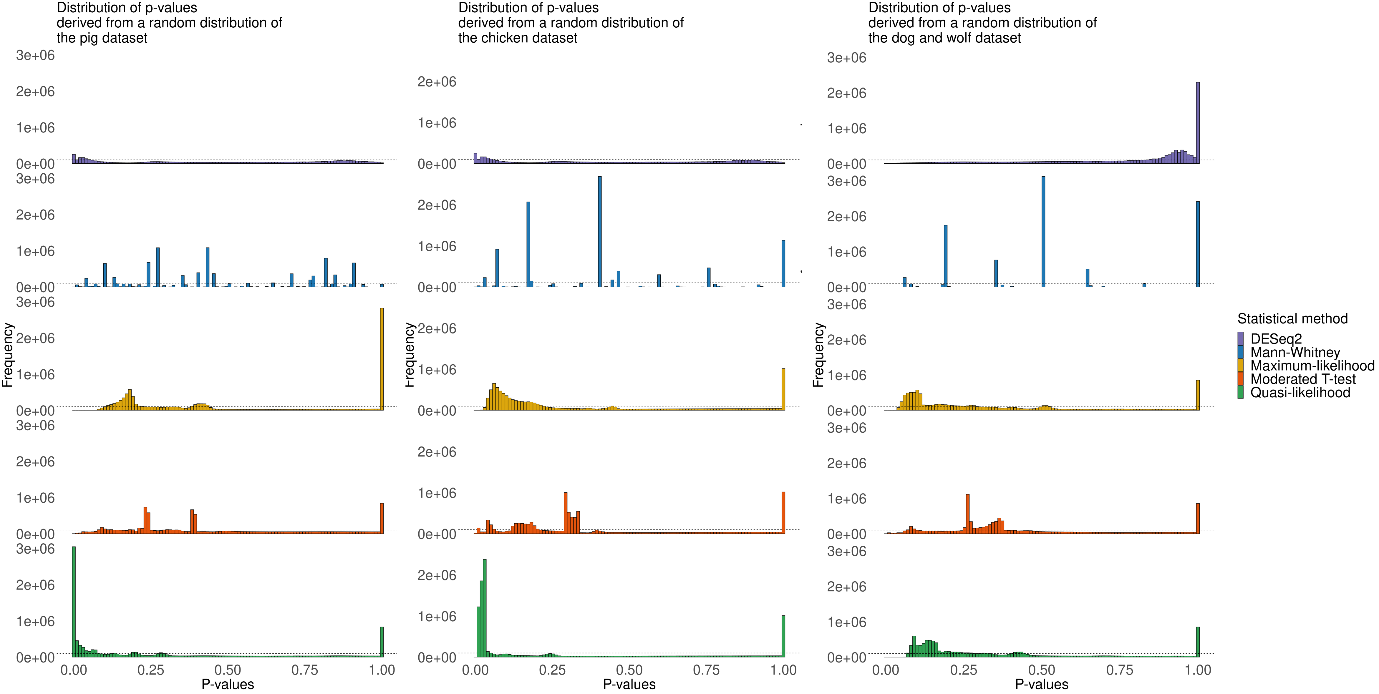


Supplementary Figure 3. Distribution of p-values derived from randomization of counts for the three datasets used in this study and each of the methods evaluated in the benchmarking. The dash line represents the uniform distribution if the p-values were derived from a random distribution and the method handles the data correctly.
